# Predicting Chronic Subdural Hematoma Resolution and Time to Resolution Following Surgical Evacuation

**DOI:** 10.3389/fneur.2020.00677

**Published:** 2020-07-14

**Authors:** Cory L. Chang, Justin L. Sim, Mychael W. Delgardo, Diana T. Ruan, E. Sander Connolly

**Affiliations:** Cerebrovascular Research Laboratory, Department of Neurological Surgery, Columbia University Irving Medical Center, New York, NY, United States

**Keywords:** chronic subdural hematoma, subdural hematoma, surgical evacuation, hematoma resolution, hematoma recurrence, traumatic brain injury, burr hole, craniotomy

## Abstract

**Background:** Growing evidence suggests that chronic subdural hematoma (CSDH) may have long-term adverse effects even after surgical evacuation. Hematoma recurrence is commonly reported as a short-term, postoperative outcome measure for CSDH, but other measures such as hematoma resolution may provide better insight regarding mechanisms behind longer-term sequelae. This study aims to characterize postoperative resolution times and identify predictors for this relatively unexplored metric.

**Methods:** Consecutive cases (*N* = 122) of burr hole evacuation for CSDH by a single neurosurgeon at Columbia University Irving Medical Center from 2000 to 2019 were retrospectively identified. Patient characteristics, presenting factors, and date of hematoma resolution were abstracted from the electronic health record. Outcome measures included CSDH resolution at 6 months, surgery-to-resolution time, and inpatient mortality. Univariate and multivariate analyses were performed to determine predictors of outcome measures.

**Results:** Hematoma resolution at 6 months was observed in 58 patients (47.5%), and median surgery-to-resolution time was 161 days (IQR: 85–367). Heavy drinking was predictive of non-resolution at 6 months and longer surgery-to-resolution time, while increased age was predictive of non-resolution at 6 months. Antiplatelet agent resumption was associated with non-resolution at 6 months and longer surgery-to-resolution time on univariate analysis but was not significant on multivariate analysis.

**Conclusion:** Postoperative resolution times for most CSDHs are on the order of several months to a year, and delayed resolution is linked to heavy drinking and advanced age. Subsequent prospective studies are needed to directly assess the utility of hematoma resolution as a potential metric for long-term functional and cognitive outcomes of CSDH.

## Introduction

Chronic subdural hematoma (CSDH) has become an increasingly common neurological condition worldwide, with an estimated incidence of up to 20.6 per 100,000 persons per year (1) and 58 per 100,000 per year for those 70 years of age and older (2, 3). While CSDH management varies based on a host of patient characteristics and radiological factors, the mainstay of treatment is neurosurgical evacuation, typically via burr hole drainage (most common), craniotomy, or craniectomy. Very few cases of CSDH resolve spontaneously (4), and even with surgery, hematoma recurrence occurs in 5–30% of reported cases (5–7). Several studies have used hematoma recurrence as a primary outcome variable and investigated predictors for this outcome (3, 7–11). The results of these analyses are quite heterogeneous, due in part to discrepancies in defining CSDH recurrence and variation in surgical and follow-up protocol (12).

In contrast, few studies have investigated predictors of CSDH resolution and CSDH resolution time—though post-operative residual fluid is detected in up to 80% of cases, it often does not precipitate acute symptoms and thus is typically deemed “clinically insignificant” (13). However, there is evidence that residual blood can have adverse effects, such as the prevention of brain re-expansion and prolongation of an inflammatory state (14, 15). While recurrence is a well-documented outcome metric for the acute follow-up period, it may not capture insidious processes that can hinder recovery and lead to poorer long-term functional and cognitive outcomes. In our single-center retrospective study, we aimed to characterize and elucidate potential predictors of CSDH resolution and surgery-to-resolution time.

## Methods

### Study Population

The study protocol was approved by the institutional review board (IRB) of Columbia University. Consecutive cases of first-time burr hole evacuation for CSDH performed by a single attending neurosurgeon at Columbia University Irving Medical Center between 2000 and 2019 were retrospectively identified. Patients who had undergone bedside hematoma evacuation procedures prior to surgical evacuation were excluded. Patients who did not have at least 6 months of post-operative follow-up (except in cases of death or where their hematoma had already resolved) were also excluded. Diagnosis of CSDH was confirmed via head computed tomography (CT) or magnetic resonance imaging (MRI). All included patients had already been discharged at the time of study initiation, and informed consent was waived by the IRB.

### Surgical Procedure

Of the 122 cases identified, 90 and 32 procedures were performed under general and local anesthesia, respectively. All patients underwent standard single- or double-burr hole craniostomy. Burr holes were created via a high-speed perforator, followed by waxing of the bone edges and opening of the dura in a cruciate fashion. The spaces were copiously irrigated with Tis-U-Sol, and titanium burr hole covers were placed over the durotomies. Closed drainage systems (Hemovac and/or Jackson-Pratt drain) were inserted in all patients and typically removed within 72 h. Antiplatelet agents and anticoagulants were discontinued prior to surgery, and reversal agents (Vitamin K for warfarin, desmopressin for antiplatelet agents) were administered as needed. These medications were re-prescribed at the discretion of the patients' providers, based on individualized consideration of both post-operative status and pre-existing comorbidities.

### Variables and Outcome Measures

Patient characteristics data including age, ethnicity, hypertension, diabetes mellitus, heavy alcohol use (>3 drinks per day or >7 drinks per week for women; >4 drinks per day or >14 drinks per week for men), history of prior SDH, recent history of head trauma, recent history of open cranial surgery, antiplatelet agent use (aspirin and/or adenosine-diphosphate receptor inhibitors) at diagnosis, and anticoagulant use (warfarin or direct oral anticoagulants) at diagnosis were collected from the electronic health record (EHR). Radiological factors including laterality and density of CSDH were recorded both at diagnosis and immediately prior to surgery. Number of burr holes, year of surgery (representing attending surgeon experience), month of surgery normalized to the academic year (representing resident experience), diagnosis-to-surgery time, length of hospitalization, and discharge modified Rankin Scale (mRS) were also noted.

Follow-up radiological examinations were conducted routinely within the first 24 h post-operatively as well as prior to discharge. Additional scans were selectively conducted for patients with large residual hematomas, changes in neurological status, and/or as part of outpatient follow-up. The mean number of CT and MRI scans obtained for each patient was 6.2 and 0.3, respectively. Short-term imaging outcomes (i.e., on last CT/MRI prior to discharge) including stability of residual hematoma, ventricular dilation, residual mass effect, and pneumocephalus were recorded, as well as time to last radiological follow-up and hematoma recurrence. Hematoma recurrence was defined as radiologically-confirmed reaccumulation of SDH in the ipsilateral subdural space during the postoperative follow-up period, causing neurological deficits that necessitated repeat surgical evacuation. Primary outcome measures included hematoma resolution at 6 months, surgery-to-resolution time, and mortality prior to discharge. Hematoma resolution was defined as disappearance of CSDH on head CT or MRI by the last follow-up scan.

### Statistical Analysis

Statistical analysis was performed via Stata/IC (Version 16, StataCorp), and identifiable personal health information was removed prior to analysis. Data are presented as mean ± standard deviation (SD) or median (interquartile range [IQR]) for continuous variables and frequency (percentage) for categorical variables. Univariate analyses were conducted via Student's *t*-test (or Welch's *t*-test for heteroscedastic data) for continuous-to-categorical comparisons, Spearman rank correlation for continuous-to-continuous comparisons, and simple logistic regression for categorical-to-categorical comparisons. Continuous variables with skewed distributions were log-transformed to achieve normality. Factors predictive in univariate analyses (*p* < 0.25) were entered into multivariate analyses (after further culling to minimize multicollinearity), carried out via multiple logistic regression, Firth's logistic regression, or multiple regression as appropriate. *P* < 0.05 was considered statistically significant.

## Results

### Patient Characteristics and Outcomes

A total of 122 patients were included in this study, and their characteristics and outcomes are detailed in Table 1. The mean age was 73.6 ± 11.6 years (range: 36–95 years), and 39 (32.0%) patients were female. Common comorbidities included hypertension (73.0%), diabetes mellitus (28.7%), and history of heavy drinking (7.4%). At the time of CSDH diagnosis, antiplatelet agents were used in 51 (41.8%) patients (86.3% of which were on aspirin) and anticoagulants were used in 20 (16.4%) patients (60.0% of which were on warfarin). Antiplatelet agents were restarted in 12 patients (9.8%) a median of 22.5 days (IQR: 14–70.5) post-operatively, and anticoagulants were restarted in eight patients (6.6%) a median of 28 days (IQR: 8.5–63) post-operatively. Forty-three (35.2%) patients had bilateral hematomas, and 105 (86.1%) had mixed-density hematomas.

**Table 1:** Patient characteristics, presenting factors, procedure types, and outcomes.

**Age, mean ± SD**	73.6 ± 11.6
Female, *n* (%)	39 (32.0)
Hispanic ethnicity, *n* (%)	50 (41.0)
**Comorbidities, *n* (%)**	
Prior subdural hematoma	9 (7.4)
Hypertension	89 (73.0)
Diabetes mellitus	35 (28.7)
Heavy drinking	9 (7.4)
Antiplatelet agent use at time of diagnosis	51 (41.8)
Antiplatelet agent use after evacuation	12 (9.8)
Surgery-to-antiplatelet agent resumption time, *d*, median (IQR)	22.5 (14–70.5)
Anticoagulant use at time of diagnosis	20 (16.4)
Anticoagulant use after evacuation	8 (6.6)
Surgery-to-anticoagulant resumption time, *d*, median (IQR)	28 (8.5–63)
**Pre-operative factors, *n* (%)**	
Recent head trauma	73 (59.8)
Recent open cranial surgery	8 (6.6)
Bilateral SDH	43 (35.2)
Mixed density SDH	105 (86.1)
**Procedure type, *n* (%)**	
Single burr hole	20 (16.4)
Double burr hole	102 (83.6)
**Short-term imaging outcomes (last prior to discharge), *n* (%)**	
Stable residual collection	78 (63.9)
Decreasing residual collection	21 (17.2)
Ventricular dilation	8 (6.6)
Residual mass effect	99 (81.1)
Pneumocephalus	100 (82.0)
Diagnosis-to-surgery time, *d*, median (IQR)	2 (1–6)
Length of hospitalization, *d*, median (IQR)	7.5 (5–13)
Surgery-to-last-follow-up time, *d*, median (IQR)	166.5 (79–408)
Discharge modified Rankin Scale, median (IQR)	3 (2–4)
Hematoma recurrence, *n* (%)	14 (11.5)
Hematoma resolution, *n* (%)	98 (80.3)
Hematoma resolution at 6 months, *n* (%)	58 (47.5)
Surgery-to-resolution time, *d*, median (IQR)	161 (85–367)
Mortality prior to discharge, *n* (%)	7 (5.7)

Single burr-hole craniostomy was performed in 20 (16.4%) patients and double burr-hole craniostomy in 102 (83.6%) patients. Median diagnosis-to-surgery time was 2 days (IQR: 1–6), median length of hospital stay was 7.5 days (IQR: 5–13), and median discharge mRS was 3 (IQR: 2–4). On the last CT/MRI prior to discharge, stable residual collection was noted in 78 (63.9%) patients, decreasing residual collection in 21 (17.2%), ventricular dilation in 8 (6.6%), residual mass effect in 99 (81.1%), and pneumocephalus in 100 (82.0%). Recurrence occurred in 14 (11.5%) patients, all of whom underwent repeat evacuations. Hematoma resolution was observed in 98 (80.3%) patients.

### Predictors of Hematoma Resolution at 6 Months and Surgery-to-Resolution Time

Hematoma resolution at 6 months was observed in 58 (47.5%) patients. Predictors of hematoma resolution at 6 months are detailed in Table 2. Increased age (OR: 0.95, 95% CI: 0.92–0.99, *p* = 0.009) and history of heavy drinking (OR: 0.18, 95% CI: 0.03–0.94, *p* = 0.042) were both found to be significantly predictive of hematoma non-resolution on multivariate analysis. Antiplatelet agent use after CSDH evacuation was predictive of non-resolution on univariate analysis (OR: 0.22, 95% CI: 0.05–0.95, *p* = 0.042) but did not reach significance on multivariate analysis. Recent head trauma (OR: 0.60, 95% CI: 0.29–1.25, *p* = 0.172) and later month of operation (normalized to the academic year; OR: 1.07, 95% CI: 0.96–1.20, *p* = 0.203) showed modest trends but were not significant on univariate analysis. No significant association was found between hematoma resolution at 6 months and presenting imaging characteristics, number of burr holes, or short-term imaging outcomes.

**Table 2:** Predictors of hematoma resolution at 6 months.

	**Odds ratio (95% CI)**	***P*-value**
**Univariate analysis**		
Age	0.96 (0.93–0.99)	**0.012**
Month of operation (normalized to academic year)	1.07 (0.96–1.20)	0.203
Recent head trauma	0.60 (0.29–1.25)	0.172
Heavy drinking	0.29 (0.06–1.46)	0.134
Antiplatelet agent use after evacuation	0.22 (0.05–0.95)	**0.042**
**Multivariate analysis**		
Age	0.95 (0.92–0.99)	**0.009**
Heavy drinking	0.18 (0.03–0.94)	**0.042**

*Predictors with p < 0.25 on univariate analysis and p < 0.05 on multivariate analysis are reported. CI, confidence interval. Bold indicates statistical significance*.

Of the patients who experienced hematoma resolution, the median surgery-to-resolution time was 161 days (IQR: 85–367 days). A subgroup analysis was performed to elucidate predictors of surgery-to-resolution time (log-transformed to account for non-normality), the results of which are summarized in Table 3 below. Heavy drinking was independently predictive of longer resolution time, with a 213.64% (95% CI: 33.67–635.93%) increase in surgery-to-resolution time (*p* = 0.009). Antiplatelet agent use was associated with longer surgery-to-resolution time on univariate analysis (ratio of geometric means: 3.15, 95% CI: 1.12–8.86, *p* = 0.036) and trended toward significance on multiple regression (*p* = 0.134). Anticoagulant use at diagnosis was not predictive on univariate or multivariate analysis, and no significant difference in resolution time was found between those who resumed anticoagulation post-operatively and those who did not. Later year of operation showed a modest trend toward shorter surgery-to-resolution time on univariate analysis (Spearman's ρ = −0.147, 95% CI:−0.336–0.052, *p* = 0.147) but was not predictive on multivariate analysis. No significant associations were found between surgery-to-resolution time and patient demographics, presenting imaging characteristics, number of burr holes, or short-term imaging outcomes.

**Table 3:** Predictors of longer surgery-to-resolution time.

	**Effect size (95% CI)**	***P*-value**
**Univariate analysis**		
Year of operation	−0.147 (−0.336–0.052)^*^	0.147
Decreasing collection	0.67 (0.35–1.27)^†^	0.226
Heavy drinking	4.67 (2.06–10.60)^†^	**<0.001**
Antiplatelet agent use after evacuation	3.15 (1.12–8.86)^†^	**0.036**
Anticoagulant use at time of diagnosis	1.58 (0.80–3.12)^†^	0.186
**Multivariate analysis**		
Heavy drinking	213.64 (33.67–635.93)^‡^	**0.009**

*Predictors with p < 0.25 on univariate analysis and p < 0.05 on multivariate analysis are reported. CI, confidence interval*.

* *Indicates Spearman's ρ*.

† *Indicates ratio of geometric means*.

‡ *Indicates percent increase in dependent variable (surgery-to-resolution time). Bold indicates statistical significance*.

### Predictors of Inpatient Mortality

Seven (5.7%) patients died prior to discharge, and predictors of inpatient mortality are summarized in Table 4. Length of hospitalization was significantly associated with higher mortality (OR: 1.03, 95% CI: 1.00–1.07, *p* = 0.047) on univariate analysis, and anticoagulant use at time of diagnosis strongly trended toward higher mortality (OR: 4.38, 95% CI: 0.99–19.36, *p* = 0.052). None of the variables investigated were found to be independently predictive on multivariate analysis. No significant associations were found between inpatient mortality and patient demographics, presenting imaging characteristics, or number of burr holes.

**Table 4:** Predictors of inpatient mortality.

	**Odds ratio (95% CI)**	***P*-value**
**Univariate analysis**		
Length of hospitalization	1.03 (1.00–1.07)	**0.047**
Hispanic ethnicity	0.31 (0.05–1.90)	0.206
Anticoagulant use at time of diagnosis	4.38 (0.99–19.36)	0.052

*Predictors with p < 0.25 on univariate analysis are reported. CI, confidence interval. Bold indicates statistical significance*.

## Discussion

Several previous studies have used hematoma recurrence as a variable when assessing CSDH post-surgical outcomes (3, 7–11), but its prognostic utility is limited primarily to the short-term follow-up period. Though given far less attention in the literature given its unclear clinical correlations, characterizing hematoma resolution may provide insight into insidious, longer-term functional and cognitive sequelae of CSDH. In the first study of long-term CSDH outcomes, patients who had undergone surgical evacuation were found to demonstrate poorer long-term functional, cognitive, and mental health outcomes when compared to matched controls, in addition to decreased long-term survival (16). CSDH survivors with dementia have also been reported to experience greater rates of brain volume loss than those without dementia, suggesting that CSDH may be accelerating the effect of brain atrophy in that population (17). None of these studies used hematoma resolution as an outcome variable (and did not report CT or MRI follow-up beyond the immediate postoperative course). Thus, though these long-term effects may be manifestations of poor baseline status (with onset of CSDH being a “sentinel” event), it is entirely possible that unresolved CSDH could contribute to such outcomes through mechanisms such as impairment of brain re-expansion or maintenance of a prolonged state of inflammation in the intracranial environment (14, 15). Brain inflammation, in particular, has been linked to neurodegenerative conditions such as Alzheimer's and Parkinson's disease, suggesting that it may play a role in cognitive dysfunction (18). Though a direct investigation of correlations between hematoma resolution and long-term outcomes is outside the scope of this present study, we believe that characterizing CSDH resolution rates and their predictors serves an important intermediate step toward that end.

In our cohort, 47.5% of patients experienced hematoma resolution by 6 months after burr hole evacuation, of which the median surgery-to-resolution time was 161 days (IQR: 85–367); these metrics are similar to those reported in other recent studies (19, 20). These results suggest that a large proportion of CSDH survivors continue to live with residual hematomas for several months or even years. As we excluded patients without at least 6 months of imaging follow-up, it is possible that a disproportionate amount of excluded cases resolved sooner than our resolution times may suggest. However, if we use length of follow-up for all consecutive burr hole cases (median: 96.5 days, IQR: 27.5–241 days) as a proxy for a best-case scenario, the time to resolution for a majority of our patients would still be on the order of multiple months or more.

Of the patient demographic factors, medical comorbidities, and presenting characteristics we investigated, history of heavy drinking was found to be predictive of CSDH non-resolution at 6 months and longer surgery-to-resolution time, and advanced age was predictive of non-resolution at 6 months. Cerebral atrophy is a well-described sequela of increased age and excessive alcohol use, which may impede brain re-expansion following CSDH and result in continued vulnerability of bridging veins (21, 22). In contrast, several other frequently reported predictors of hematoma recurrence were not predictive of resolution or resolution time in our cohort, particularly diabetes mellitus (23), bilateral hematoma (22, 24), and loculated-type hematoma (25–28). The discrepancy with regard to presenting radiological features is especially interesting, as it suggests that the nature of the hematoma itself may be of lesser importance for longer-term prognostication, perhaps as adequate surgical evacuation can ameliorate anatomical impediments to resolution posed by complex collections.

Antiplatelet agents were used by 51 (41.8%) of our patients at the time of CSDH diagnosis, of which 12 restarted antiplatelet therapy after a median of 22.5 days post-operatively. Antiplatelet agent resumption was significantly linked to both CSDH non-resolution at 6 months and longer surgery-to-resolution time on univariate analysis, but these associations only trended toward significance on multivariate analysis. Use of anticoagulants has been a well-documented risk factors for CSDH development, and several studies have reported its association with CSDH recurrence as well—however, the association between antiplatelet resumption and rebleeding remains unclear (23, 29). Our relatively small sample size for this subgroup may not have sufficient power to detect this potential effect, and larger studies are needed before a link between antiplatelet resumption to CSDH resolution (or lack thereof) can be established.

Interestingly, no significant differences in outcome were found between those who resumed anticoagulation after surgery and those who did not, though our sample size again limits the statistical power of this assessment. A meta-analysis of three studies of CSDH recurrence vs. thromboembolic risk found that all recurrences and thromboembolic events occurred within the 1st month post-operation (30). As the median time to anticoagulant reinsertion was 28 days in our cohort, there was perhaps a sufficient window of time for adequate clot formation, resulting in an insignificant difference in resolution time between patients who resumed and did not resume anticoagulation. While the feasibility of resuming early antithrombotic treatment in certain patients has been reported, there is yet no consensus on the reinsertion of therapy (30–32). Antithrombotic resumption in post-operative CSDH patients remains at the discretion of the clinician, tailored to individual patients' hemorrhagic and thromboembolic risks. Should CSDH resolution be found as a long-term clinically salient metric in future studies, extra consideration may be warranted when determining the best time interval for restarting antithrombotic therapy in these patients.

No differences in outcomes were found between single burr-hole and double burr-hole evacuation, and recent studies have also not reported any association between type of procedure and hematoma recurrence (22, 25, 33). Discharge mRS and imaging features prior to discharge (e.g., stable/decreasing collection, residual mass effect) also did not yield significance on multivariate analyses, suggesting that such short-term metrics may not be reliable predictors of CSDH resolution. Later year and month (normalized to the academic year) of surgery showed only modest univariate trends toward shorter resolution time and resolution at 6 months with minimal effect sizes. As these temporal variables were not significant on multivariate analysis, attending and resident procedural experience do not appear to be major independent contributors to our outcomes.

Our study has several limitations. All cases of CSDH evacuation were performed by a single neurosurgeon at Columbia University Irving Medical Center, which limits the external validity of our results. The retrospective nature of our study also precluded any predetermined imaging follow-up protocol, which may have introduced selection bias when excluding patients without the requisite 6 months of follow-up (e.g., perhaps the excluded patients tended to recover more quickly). Multi-center prospective, controlled studies with standardized long-term follow-up protocol will be necessary to validate and expand upon our findings.

## Conclusion

Increasing evidence suggests that CSDH survivors suffer from long-term functional and cognitive outcomes despite successful surgical evacuation. While hematoma recurrence is a commonly used metric for short-term outcomes, we hypothesize that hematoma resolution and surgery-to-resolution time may better capture such adverse effects. Our results suggest that a large proportion of CSDH patients live with residual hematomas for months or even years after surgery, and we found advanced age and history of heavy drinking to be predictive of delayed resolution. Larger prospective, controlled studies are needed to further elucidate the potential effects of antithrombotic resumption on hematoma resolution and will aim to correlate hematoma resolution with functional and cognitive outcomes.

## Data Availability Statement

The datasets generated for this study are available on request to the corresponding author.

## Ethics Statement

The studies involving human participants were reviewed and approved by Columbia University Human Research Protection Office Institutional Review Boards. Written informed consent for participation was not required for this study in accordance with the national legislation and the institutional requirements.

## Author Contributions

CC, JS, and EC contributed to the conception and design of the study. CC, JS, MD, and DR assisted in data acquisition. CC organized the database and performed the statistical analysis. CC, JS, and MD wrote the first draft of the manuscript. CC, JS, and DR contributed to manuscript revision. All authors read and approved the final version of the manuscript.

## Conflict of Interest

The authors declare that the research was conducted in the absence of any commercial or financial relationships that could be construed as a potential conflict of interest.
